# Robotic-assisted minimally invasive Ivor Lewis esophagectomy within the prospective multicenter German da Vinci Xi registry trial

**DOI:** 10.1007/s00423-022-02520-w

**Published:** 2022-05-02

**Authors:** Jan-Hendrik Egberts, Thilo Welsch, Felix Merboth, Sandra Korn, Christian Praetorius, Daniel E. Stange, Marius Distler, Matthias Biebl, Johann Pratschke, Felix Nickel, Beat Müller-Stich, Daniel Perez, Jakob R. Izbicki, Thomas Becker, Jürgen Weitz

**Affiliations:** 1 Department of General, Visceral, Thoracic, Transplantation, and Pediatric Surgery, Kurt Semm Center for Minimally Invasive and Robotic Surgery, University Hospital Schleswig Holstein, 24105 Kiel, Germany; 2 Department of Surgery, Israelitisches Krankenhaus Hamburg, 22297 Hamburg, Germany; 3 Department of Visceral, Thoracic and Vascular Surgery, University Hospital Carl Gustav Carus, Technische Universität Dresden, 01307 Dresden, Germany; 4 National Center for Tumor Diseases (NCT/UCC), Dresden, Germany: German Cancer Research Center (DKFZ), Heidelberg, Germany; Faculty of Medicine and University Hospital Carl Gustav Carus, Technische Universität Dresden, Dresden, Germany; Helmholtz-Zentrum Dresden - Rossendorf (HZDR), 01307 Dresden, Germany; 5 Department of Surgery, Charité University Hospital, 13353 Berlin, Germany; 6 Department of General, Visceral and Transplantation Surgery, University of Heidelberg, 69120 Heidelberg, Germany; 7 Department of General, Visceral and Thoracic Surgery, University Medical Center Hamburg-Eppendorf, 20246 Hamburg, Germany

**Keywords:** RAMIE, Multicenter, Ivor Lewis, Esophagectomy, Leaning curve, CUSUM analysis

## Abstract

**Abstract:**

**Purpose:**

Robotic-assisted minimally invasive esophagectomy (RAMIE) has become one standard approach for the operative treatment of esophageal tumors at specialized centers. Here, we report the results of a prospective multicenter registry for standardized RAMIE.

**Methods:**

The German da Vinci Xi registry trial included all consecutive patients who underwent RAMIE at five tertiary university centers between Oct 17, 2017, and Jun 5, 2020. RAMIE was performed according to a standard technique using an intrathoracic circular stapled esophagogastrostomy.

**Results:**

A total of 220 patients were included. The median age was 64 years. Total minimally invasive RAMIE was accomplished in 85.9%; hybrid resection with robotic-assisted thoracic approach was accomplished in an additional 11.4%. A circular stapler size of ≥28 mm was used in 84%, and the median blood loss and operative time were 200 (IQR: 80–400) ml and 425 (IQR: 335–527) min, respectively. The rate of anastomotic leakage was 13.2% (*n*=29), whereas the two centers with >70 cases each had rates of 7.0% and 12.0%. Pneumonia occurred in 19.5% of patients, and the 90-day mortality was 3.6%. Cumulative sum analysis of the operative time indicated the end of the learning curve after 22 cases.

**Conclusions:**

High-quality multicenter registry data confirm that RAMIE is a safe procedure and can be reproduced with acceptable leak rates in a multicenter setting. The learning curve is comparably low for experienced robotic surgeons.

**Supplementary Information:**

The online version contains supplementary material available at 10.1007/s00423-022-02520-w.

## Introduction

Currently, esophagectomy within a multimodal treatment plan is the preferred management of patients with resectable esophageal cancer [16]. The introduction of minimally invasive techniques for esophagectomy has revolutionized surgical treatment leading to lower perioperative morbidity and better quality of life [18, 21, 26]. Hybrid laparoscopic/thoracoscopic minimally invasive esophagectomy (MIE) and robotic-assisted minimally invasive esophagectomy (RAMIE) have both led to a significant reduction in pulmonary infections and postoperative pain in randomized clinical trials [2, 19, 34] while maintaining oncologic radicality [24]. Some key advantages of the robotic-assisted technique, especially during transthoracic resection and reconstruction, are an increased range of motion of the instruments within the rigid thoracic cage, the optional use of three arms, and an improved surgical view with standard 3DHD visualization [13]. Although several techniques of reconstruction after MIE have been reported, the majority of European centers favor minimally invasive intrathoracic esophagogastrostomy [14, 35]. The use of a circular stapler appears to be advantageous with regard to the AL rate, although this question has not yet been conclusively clarified [5]. Experienced centers have published the first larger single-center reports of RAMIE with excellent oncological results and low mortality rates of 1–3% [25, 33].

The German da Vinci Xi Registry trial was set up in 2017 in five tertiary German university centers. The aim was multicenter prospectively monitored data collection to assess the outcomes of robotic-assisted abdominal and thoracic surgery. After individual experiences in the initial period, the centers agreed on a basic consensus technique for RAMIE, which is based on an Ivor Lewis reconstruction with intrathoracic circular stapled end-to-side esophagogastrostomy [7].

The aim of the present study was to demonstrate the safety, learning curve, and short-term results of this prospective multicenter RAMIE program.

## Patients and methods

### Study design

The study was designed as a multicenter prospective registry investigator-initiated trial. Five German university centers at Berlin, Dresden, Hamburg, Heidelberg, and Kiel participated in the prospective German da Vinci Xi Registry trial. The trial was in compliance with the ethical principles of Helsinki, and the protocol was approved by the responsible independent ethics committees of all participating centers, i.e., the local ethics committee at TU Dresden (EK296072017), Christian Albrechts University in Kiel (AZD421/13, D451/19), Heidelberg University Faculty of Medicine (S-341/2017), Charité University Hospital in Berlin (EA4/084/17) and University Medical Center Hamburg-Eppendorf (PV5591).

The study was supported with a research grant by Intuitive (Sunnyvale, CA, US).

### Patients

All consecutive patients who underwent elective RAMIE with an intrathoracic circular stapled end-to-side esophagogastrostomy at each study site between Oct 15, 2017, and Jun 5, 2020, were considered for inclusion if they met the following criteria: age ≥18 years and written informed consent. All patients with esophageal cancer or an indication for esophagectomy who were suitable for a minimally invasive approach using gastric tube reconstruction were considered for the primary robotic-assisted minimally invasive approach. There was no standard selection criteria for the avoidance of the robotic approach. The da Vinci Xi robotic surgical systems were available for all scheduled patients without limitations. One center was delayed in starting RAMIE because of a concurring randomized trial of open versus total minimally invasive laparothoracoscopic esophagectomy. Surgeons’ and patients’ preferences as well as availability of the da Vinci robotic system were taken into account regarding the choice of the procedure in the latter center. The exclusion criteria were emergency operations, patients with a survival prognosis of less than 1 month, operations for which the da Vinci Xi system was not approved (according to the manufacturer), and pregnancy. The study was managed and monitored by the local study centers at the participating sites. Intraoperative documentation was performed by authorized senior surgeons.

### Surgical technique

The basic steps of the surgical technique used for all operations were published recently [7, 9]. After such consensus of the basic surgical steps was obtained, a proctoring during initial operations was conducted by the most experienced surgeons [14]. All operations were performed on a da Vinci Xi surgical system (Intuitive, Sunnyvale, CA, US). Briefly, the patients were placed in a supine and 15°–20° reverse Trendelenburg position for the abdominal part. The four da Vinci Xi trocars were inserted on a horizontal line usually above the umbilicus supplemented with one additional assistant trocar. Lymphadenectomy included lymph node stations along the hepatic and splenic arteries centrally toward the left gastric artery and the celiac trunk. The left gastric vessels were divided using clips, and the lymph node package ventral to the aorta at the hiatus was resected *en bloc* with the esophagogastric junction. The gastric tube was trimmed to a semicircumference of 45 mm using linear stapling devices 45 or 60 mm in length beginning at the angular notch. The semicircumference of 45 mm was calculated to allow a circular anastomosis with a 28-–29-mm diameter. Indocyanine green (ICG) fluorescence analysis was routinely performed to define the optimal perfusion margin of the gastric tube. For the thoracic part, the patients were turned into a left lateral to semiprone position, and the right lung was not ventilated. The four da Vinci Xi trocars were placed in a banana-shaped fashion between the fourth and tenth intercostal spaces of the right hemithorax. One additional assistant trocar was used. After docking, the azygos vein was divided, and the esophagus was dissected, including the adjacent paraaortal and mediastinal lymph node stations. The thoracic duct was identified and clipped. If preoperative imaging analysis indicated lymph node metastases along the recurrent nerves, an extended paratracheal lymphadenectomy was performed, e.g., lymph node level two. After complete resection, the esophagus was divided and closed with a purse-string suture. The proximal resection margin of the esophagus was assessed by intraoperative frozen section analysis. At this point, the esophageal resection specimen was extracted using a minithoracotomy at a trocar site, and the stapler anvil was inserted into the oral esophageal stump. Consecutively, the end-to-side esophagogastrostomy was stapled through the minithoracotomy, and the proximal part of the stomach was stapled 2 cm from the esophagogastrostomy using a linear stapling device. The esophagogastrostomy anastomosis was reinforced using either a robotic-assisted running suture or an omental wrap or both. A nasogastric tube was intraoperatively placed distally to the esophagogastrostomy, and one to two chest tubes were placed according to the SOP at each center at the time of operation.

### Definition of postoperative morbidity

Postoperative morbidity was assessed using the classification of postoperative complications according to Clavien-Dindo [4, 6] with substantiation by the “Japan Clinical Oncology Group” [12]. The following complications were considered: anastomotic leak (AL), pneumonia, respiratory insufficiency, readmission to the intensive care unit (ICU), disorder of gastrointestinal passage, recurrent laryngeal nerve palsy, cardiac arrhythmia, chylothorax, postoperative hemorrhage, wound dehiscence, surgical site infection, intrathoracic fluid collections, mediastinitis, empyema/pyothorax, thromboembolic events, acute renal failure, cardiac decompensation, bacteremia/sepsis, and multiple organ failure.

Based on the recommendations of the Esophagectomy Complications Consensus Group (ECCG), AL was defined as a full-thickness gastrointestinal defect involving the esophagus, anastomosis, stapler line, or conduit irrespective of presentation or method of identification [17]. Diagnosis of AL was made on the basis of contrast leakage on CT, endoscopically or both. For the definition of pneumonia, at least one major and two minor criteria had to be fulfilled (major: new infiltrate in chest imaging; minor: fever >38.5°C or hypothermia <36.5°C, new elevation or permanent high infection markers (leucocytes/C-reactive protein/procalcitonin), productive cough with sputum, pathogen detection) [11].

Textbook outcome was defined as R0 resection with no conversion, a lymph node yield ≥15, no complications of Clavien-Dindo ≥3a, no reinterventions or reoperations, no readmission to the ICU, length of hospital stay ≤21 days, no hospital readmission, and no mortality within 90 days postoperatively [14].

### Statistical analysis

The SPSS (version 27.0.0.0) software package was used for statistical calculation and data plots. The significance level for all calculations was set at *p*=0.05. The operative time was defined from the start of the operative procedure with a skin incision at the abdomen until skin suturing of the thoracic part, including docking and repositioning times. For learning curve analysis, a subgrouping of 15 consecutive patients was performed for each center. Moreover, we accomplished a case grouping (*n*=15) for each surgeon with more than 30 cases and studied the median reduction in the operative time of subsequent cases compared with the initial 15 cases. For further investigation of the learning curve, a cumulative sum (CUSUM) analysis of the total operative time was performed. This technique is a graphical method to transform raw data into a running total of differences from the group average. Therefore, a chronological arrangement of all cases from the first to the last by the center (or by the leading surgeon, respectively) was performed. Then, CUSUM values were calculated according to the following formula: CUSUM = *Σ* (*x*_i_−*μ*), where *x*_i_ is the total operative time of the individual case and *μ* is the mean operative time of the corresponding center or leading surgeon [30, 36]. Finally, the CUSUM values were plotted on the vertical axis according to their case number on the horizontal axis. Learning curves could be determined by visual interpretation of the chart. The end of the learning curve was predefined as inflection of the curve to a plateau or decrease.

Continuous variables are presented as medians with interquartile ranges (IQRs). The evaluation for nonparametric variables was performed with the Mann-Whitney *U* test. Univariate analysis was computed using cross tabulation and the chi-square test or Fisher’s exact test.

## Results

### Patient characteristics and histopathological results

In total, 220 patients were included in the analysis (center 1: 72; center 2: 41; center 3: 83; center 4: 10; center 5: 14). The median age of the patients was 64 years (IQR 58–72), and 85.5% (*n*=188) were male. Two-thirds of the patients had significant comorbidities (ASA ≥III), and the median BMI was 26.2 kg/m^2^ (IQR 23.6–29.4). No further information of race or ethnicity of the patients was collected routinely in the Xi trial. The indications for esophagectomy were adenocarcinoma in 81.4%, squamous cell carcinoma in 15.5% and other diseases (malignant and nonmalignant) in 3.2% of the cases. Most of the patients had neoadjuvant treatment, including chemoradiation (32.7%) or chemotherapy (47.7%) (Table 1). The distribution of pTNM stage and UICC stage is shown in Table 1.

**Table 1:** Patient characteristics and pathological findings (n=220)

	***n*** **/median** **(%/IQR)**
**Age [years]**	64 (58-72)
**Sex**	
Female	32 (14.5)
Male	188 (85.5)
**BMI [kg/m**^**2**^**]**	26.2 (23.6-29.4)
**ASA**	
1	4 (1.8)
2	70 (32.3)
3	141 (65.0)
4	2 (0.9)
**Histology**	
Adenocarcinoma	179 (81.4)
Squamous carcinoma	34 (15.5)
Other	7 (3.2)
**Neoadjuvant treatment**	
Chemotherapy	105 (47.7)
Chemoradiation	72 (32.7)
None	43 (19.5)
**pT stage**	
0	32 (15.0)
1	46 (21.6)
2	42 (19.7)
3	90 (42.3)
4	3 (1.4)
**pN stage**	
0	120 (56.6)
1	48 (22.6)
2	30 (14.2)
3	14 (6.6)
**pM stage**	
0	206 (96.7)
1	7 (3.3)
**UICC stage**	
I	82 (38.5)
II	33 (15.5)
III	77 (36.1)
IV	21 (9.9)

*BMI* body mass index, *ASA* American Society of Anesthesiologists, *n (%)* median (IQR)

### Surgical technique

A totally robotic-assisted operation of both the abdominal and thoracic part (RAMIE) was accomplished in 189 cases (85.9%), whereas a hybrid minimally invasive approach (hRAMIE) with a robotic thoracic part and open abdominal part was performed in 25 cases (11.4%). A hybrid robotic abdominal operation with open thoracotomy was performed in 6 cases (2.7%). Laparoscopy or thoracoscopy was not used alternatively for hybrid approaches. The main reasons for a hybrid approach were a learning phase strategy (*n*=14), extended dissection for lymph node metastasis (*n*=4), and adhesions/prior surgery (*n*=4). Overall conversion to an open procedure was necessary in 16 cases (7.3%). The most frequent reasons for conversion were adhesions (*n*=5) and intraoperative bleeding (*n*=4). Extended thoracic resection because of lung infiltration was necessary in 7 cases. One center routinely placed jejunal feeding tubes during the abdominal part. A circular stapler size of ≥28 mm was used in most cases (84%). The median blood loss was 200 ml (IQR 80–400), and the median operative time (OT) was 425 min (IQR 335–527) (Table 2).

**Table 2:** Surgical findings (*n*=220)

	***n*** **/median** **(%/IQR)**
**Surgical technique**	
RAMIE (abd. + tho. rob.)	189 (85.9)
Conversion (tho.)	8 (4.2)
Conversion (abd.)	6 (3.2)
hRAMIE (only tho. rob.)	25 (11.4)
Conversion (tho.)	1 (4.0)
hRAMIE (only abd. rob.)	6 (2.7)
Conversion (abd.)	1 (16.7)
**Reasons for hybrid procedure**	
Approach learning phase	14
Extended lymphadenectomy	4
Adhesions/former surgery	4
Infiltration of adjacent structures	3
Tumor bleeding	2
Others	4
**Reasons for conversion**	
Adhesions	5
Bleeding	4
Situs	2
Technical problems	2
Others	3
**Extended** **l** **ung** **resection**	7 (3.2)
Wedge	6 (85.7)
Lobe	1 (14.3)
**Extended l** **ymphadenectomy**	100 (45.5)
Cervical	1 (1.0)
Mediastinal region 2–4	99 (99.0)
**Simultaneous jejunostomy feeding tube**	75 (34.1)
**Stapler size esophagogastrostomy**	
25 mm	34 (15.5)
28 mm	81 (37.0)
29 mm	103 (47.0)
33 mm	1 (0.5)
**Blood loss [ml]**	200 (80–400)
**Operative time [min]**	425 (335–527)
**R status**	
0	196 (92.9)
1	15 (7.1)
**Resected lymph nodes**	25 (19–30)

*RAMIE* robot-assisted minimally invasive esophagectomy, *hRAMIE* hybrid RAMIE, *abd.* abdomen, *tho.* thorax, *rob.* robotic-assisted, *n (%)* median (IQR)

Oncological resection with microscopically tumor-free margins (R0) was achieved in 92.9% of patients. Reresection because of a tumor-infiltrated resection margin reported by the intraoperative frozen section examination (which was available for all cases) was required in only two cases. The median number of resected lymph nodes was 25 (IQR 19–30) (Table 2).

### Postoperative short-term outcome

**Fig. 1:**
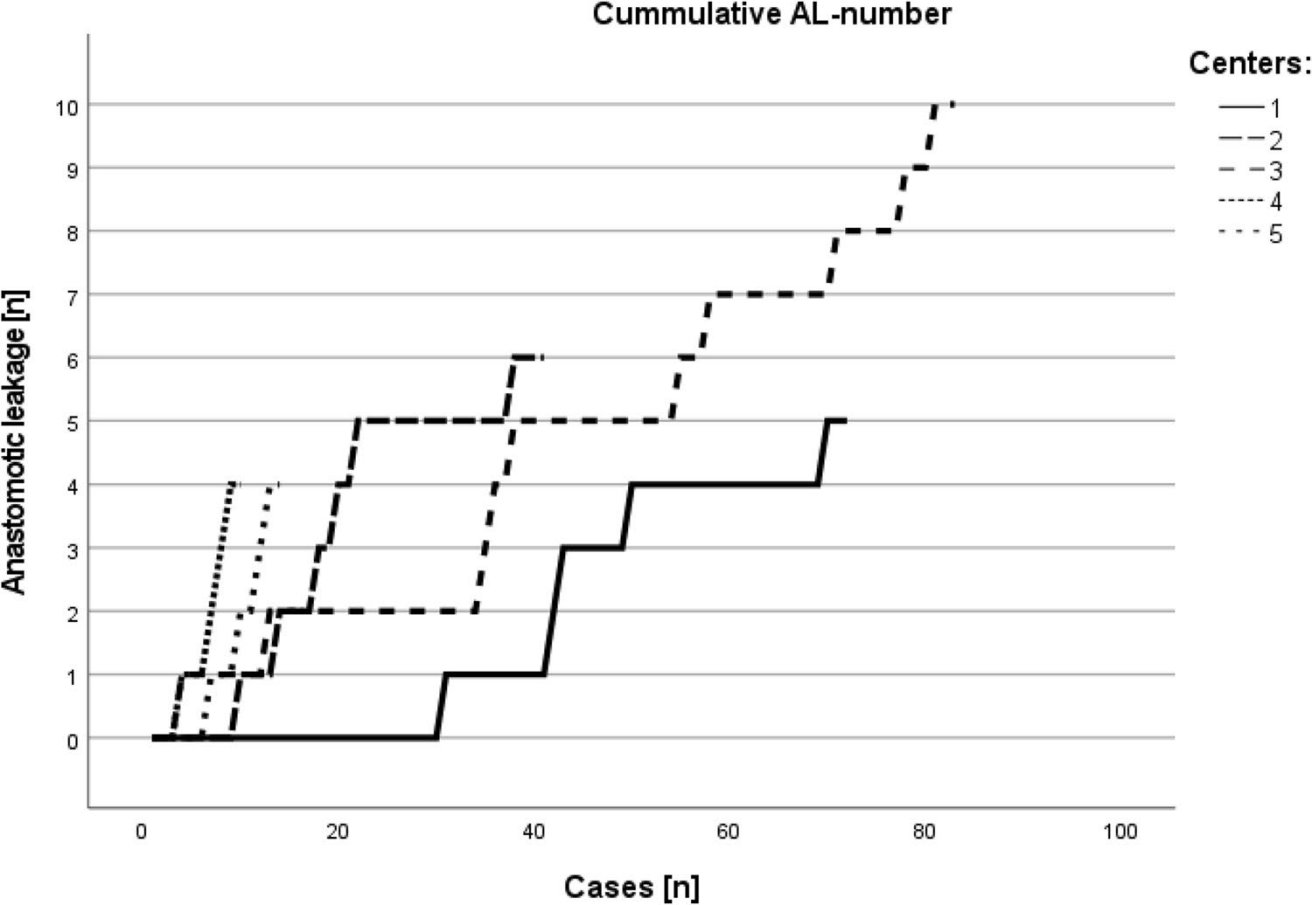
Cumulative occurrence of anastomotic leakage per case number stratified by center

Twenty-one percent (*n*=46 patients) of the total cohort developed major postoperative complications (CDC grade ≥3b) (Table 3). The rate of postoperative anastomotic leakage was 13.2% (*n*=29). Most patients (82.8%; *n*=24) with AL were successfully treated using endoluminal approaches (predominantly endoluminal vacuum therapy), whereas reoperation was indicated in 5 cases. Thereby in most cases, an esophageal diversion as well as an insertion of a jejunal feeding tube was performed. Furthermore, 27 patients underwent postoperative endoscopic interventions for indications other than AL. The rate of postoperative AL differed between the centers, and the two centers with >70 cases had leak rates of 7.0% and 12.0% (*p*=0.213), respectively (Fig. 1).

**Table 3:** Morbidity and mortality (*n*=220)

	**n/median** **(%/IQR)**
**Morbidity CDC ≥ 3b**	27 (12.3)
**Anastomotic leak**	29 (13.2)
**Treatment of leak**	
Endoscopic	24 (82.8)
Reoperation	5 (17.2)
**Pneumonia**	43 (19.5)
**Reoperation**	24 (10.9)
Minimally invasive	9 (37.5)
Open	15 (62.5)
**Reasons for reoperation**	
Anastomotic leak	5
Chylothorax	3
Ileus	3
Ischemia of gastric conduit	2
Pulmonary air leak	2
Bleeding	2
Others	7
**Postoperative endoscopic intervention**	27 (12.4)
**ICU stay**	
Postoperative [days]	2 (1-4.7)
In total [days]	3 (1-5.8)
Readmission	30 (13.6)
**Postoperative hospital stay [days]**	15 (12-24)
**90-day readmission**	18 (8.2)
**90-day mortality**	8 (3.6)
**Textbook outcome**	67 (31.6)

*CDC* Clavien-Dindo Classification, *ICU* intensive care unit, *n (%)* median (IQR)

Postoperative pneumonia was diagnosed in 19.5% of cases. A reoperation, either minimally invasive (*n*=9) or open (*n*=15), was indicated in 5 cases because of AL (including esophagobronchial fistula), chylothorax (*n*=3), postoperative ileus (*n*=3), pulmonary air leak (*n*=2), or ischemia of the gastric tube (*n*=2) (Table 3).

The median postoperative intensive care unit stay was 2 days (IQR 1–4.7), and the median hospital stay was 15 days (IQR 12–24). The 90-day readmission and mortality rates were 8.2% and 3.6%, respectively. The causes of death were multiorgan failure (*n*=3), aspiration pneumonia (*n*=1), cardiac tamponade (*n*=1), hemorrhagic shock (*n*=1), myocardial infarction (*n*=1), and tumor progression (*n*=1).

Overall, 67 patients (31.6%) had a defined textbook outcome with an optimal intra- and postoperative course.

### The impact of the learning curve on operative time

**Fig. 2:**
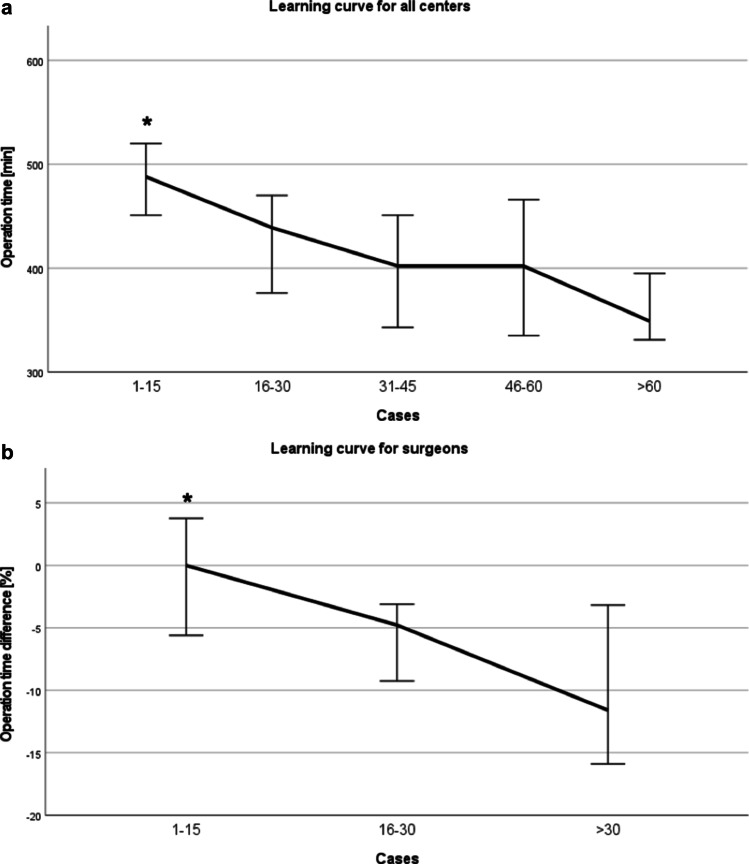
Operative time of the RAMIE procedure. **A** Operative time including abdominal and thoracic parts of all procedures in all 5 centers (*n*=220). The median operative time (min) is shown stratified by chronological grouping of 15 cases (*p≤0.024). **B** Operative time for the three surgeons with >30 RAMIE procedures. The graph displays the median difference in the operative time from the first 15 cases compared with cases 16–30 and cases >30 for the three surgeons with the highest case load (*p≤0.021)

The median total operative time within the first 15 cases of each center (*n*=69) was 488 min, which was significantly longer than that in the consecutive case groupings (*p*=0.024). The median operative time subsequently dropped from 439 min (cases 16–30; *n*=45) to 402 min (cases 31–45; *n*=41) (*p*=0.126). Although the median operative time was further reduced to 349 min after >60 cases (*n*=35), the difference was not statistically significant when compared with cases 46–60 (*p*=0.089; *n*=30) (Fig. 2a). Similar results were observed if the median operative time of the thoracic part only was analyzed: the median operative time of cases 1–15 was longer than that of the consecutive case groupings (*p*=0.023). However, a significant reduction in the operative time for the abdominal part was not seen until a caseload >60 (*p*≤0.001) (Suppl. Fig. 1). The three most experienced surgeons of the participating centers could significantly improve their individual median operative time by approximately −4.8% after cases 16–30 (*n*=45) and approximately −11.6% after >30 cases (*n=*50) (*p*≤0.021). Likewise, there was a trend toward a further operative time reduction from cases 16–30 to cases >30, but without statistical significance (*p*=0.057) (Fig. 2b).

**Fig. 3:**
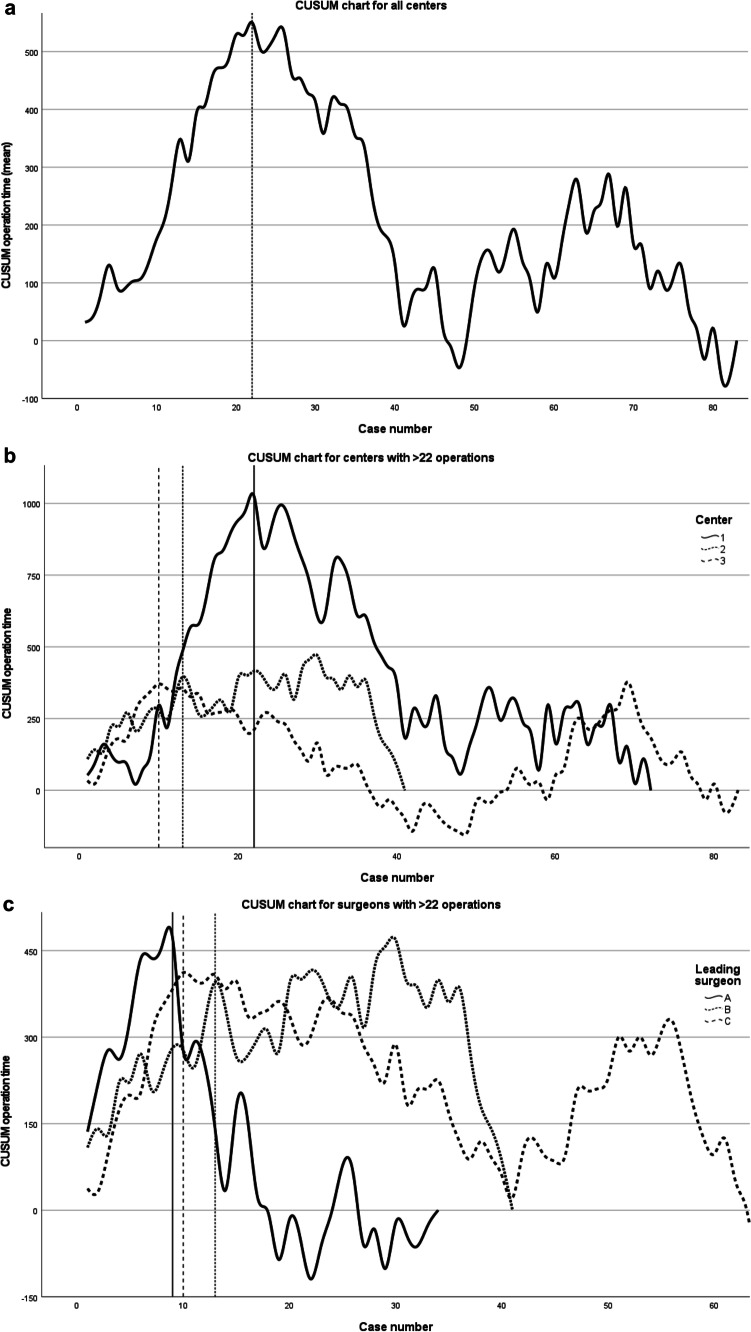
CUSUM analysis of the operative time. **A** CUSUM analysis including all five centers. The inflection point after the 22nd procedure marks the end of the learning curve. **B** CUSUM analysis including the three centers with >22 RAMIE cases. **C** The CUSUM analysis for the leading surgeons of the three most experienced centers

The pooled CUSUM graph for all centers showed a peak (inflection point) with a slow decrease after 22 cases, indicating the end of the learning curve for the total operative time (Fig. 3a). The CUSUM graphs for the three centers with more than 22 RAMIE procedures revealed different end points of the learning curve: the inflection point in center 1 was at 22 cases, center 2 reached a plateau after 13 cases, and center 3 reached a plateau after just 10 cases (Fig. 3b). The CUSUM analysis for the leading surgeons of the three most experienced centers showed the same end points of the learning curve of surgeons B and C as for their related centers 2 and 3 (Fig. 3c). This finding is not surprising because the leading surgeon in these two centers performed (nearly) all procedures (78.3% and 100%, respectively). In center 1, three surgeons routinely performed RAMIE, which explains the longer learning curve for this center. The point of inflection for leading surgeon A of center 1 was at the 9th case (Fig. 3c).

### The impact of the learning curve on intraoperative findings, postoperative course, and mortality

Based on the CUSUM analysis, perioperative outcome parameters were compared in relation to the pre- and post-learning curve cohort (≤22 and >22 cases). The latter cohort was operated on with less blood loss (*p*<0.001), a shorter operative time (*p*<0.001), and a lower rate of postoperative pneumonia (*p*=0.046). Additionally, there was a trend toward a lower conversion (11.1 to 4.6%; *p*=0.061) and 90-day readmission rate (12.2to 5.4%, *p*=0.059) after >22 cases. Other outcome parameters, including major complications CDC ≥3b, AL rate, textbook outcome, and intensive care parameters, were not significantly different between the two groups (Table 4).

**Table 4:** Intra- and postoperative findings dependent on case number (*n*=220)

	**cases 1-22** ***n*** **=90 (%/IQR)**	**cases >22** ***n*** **=130 (%/IQR)**	**OR**	**95% CI**	***p*** **-value**
**RAMIE**	69 (76.7)	120 (92.3)	**3.65**	**1.626–8.203**	**0.001**
**Conversion**	10 (11.1)	6 (4.6)	0.39	0.135–1.107	0.061
**Blood loss [ml]**	300 (127–400)	100 (40–300)	**0.99**	**0.998-1.000**	**<0.001** ^#^
**Operative time [min]**	487 (374–554)	393 (327–481)	**0.99**	**0.992-0.997**	**<0.001** ^#^
**Resected lymph nodes [n]**	25 (19–30)	24 (17–30)	0.99	0.971–1.024	0.329^#^
**Textbook outcome**	30 (34.1)	37 (29.8)	0.82	0.458–1.476	0.306
**CDC ≥ 3b**	9 (10.0)	18 (13.8)	1.45	0.618–3.383	0.261
**Anastomotic leak**	15 (16.7)	14 (10.8)	0.60	0.275–1.322	0.143
**Pneumonia**	23 (25.6)	20 (15.4)	0.53	0.271–1.037	**0.046**
**Reoperation**	8 (9.0)	16 (12.3)	1.42	0.581–3.479	0.294
**ICU stay**					
Postoperative [d]	2.0 (1.0–5.0)	2.8 (1–4.3)	1.01	0.970–1.046	0.498^#^
In total [d]	2.9 (1.0–6.0)	3.0 (1.0–5.7)	1.01	0.978–1.036	0.408^#^
Readmission	15 (16.7)	15 (11.5)	0.65	0.301–1.412	0.186
**Hospital stay [d]**	14 (12–27)	15 (12–23)	0.99	0.974–1.003	0.417^#^
**90-day readmission**	11 (12.2)	7 (5.4)	0.41	0.152–1.099	0.059
**90-day mortality**	1 (1.1)	7 (5.4)	5.07	0.612–41.902	0.093

*CDC* Clavien-Dindo Classification, *ICU* intensive care unit, *OR* odds ratio, *95% CI* 95% confidence interval, *n (%)* median (IQR), Fisher’s exact test, ^#^ Mann-Whitney *U*

All operated cases (*n*=220) were included in a univariate analysis to identify predictive factors for the development of AL (Suppl. Tab. S1). However, none of the tested variables significantly correlated with the occurrence of AL.

## Discussion

This is the first report of a prospective multicenter registry trial evaluating the short-term outcome of RAMIE with an intrathoracic circular stapled anastomosis. Because of the potentially beneficial effects of the RAMIE approach on short-term patient outcome, the participating university centers agreed on a prospective multicentric registry study to evaluate the safety and potential benefits of RAMIE during the implementation phase and beyond with a standardized technique. The aim of this registry study was to generate data to assess the da Vinci Xi surgical system for esophagectomy regarding clinical outcome. The multicenter RAMIE program included a uniform technique with an intrathoracic (Ivor Lewis) circular stapled esophagogastrostomy using a minithoracotomy. According to the present knowledge, circular stapled anastomosis is the most frequently performed anastomosis technique during RAMIE [14].

Overall, approximately 80% of the operations were minimally invasive using the da Vinci Xi robotic system (fully robotic), and the thoracic part, including the anastomosis, was robotic-assisted in 94% (207) of the cases; whereas in thoracoscopic approaches (MIE), the conversion rate was 14% [2, 28]. This result demonstrates that the RAMIE technique is feasible in most cases. The rate of a fully robotic-assisted approach was higher than that in a recent international registry report, where only 54% of the cases were not hybrid procedures [14]. Other high-volume centers for RAMIE combine an abdominal open or laparoscopic part with the robotic-assisted thoracic part [25]. A direct comparison of an open abdominal operation phase and a total RAMIE revealed no significant differences regarding oncological radicality and recurrence-free survival, suggesting that robotic-assisted abdominal lymphadenectomy is adequate [23]. The oncological quality in the present study, as indicated by the R0 resection rate (93%) and the median number of resected lymph nodes (*n*=25), is comparable with recent single-center series [14, 25, 34].

The current results from leading esophagus surgery centers support the use of a circular stapler esophagogastrostomy, especially for minimally invasive intrathoracic anastomosis [5, 22]. The stapler diameter should be selected according to the individual anatomical situation of the patient but with preference for the largest possible diameter; however, a significant difference regarding anastomotic leakage and stricture was not identified between 25- and 28-mm diameter sizes [29]. In our analysis, in 84% of all cases, a stapler size equal to or greater than 28 mm was used for esophagogastrostomy, which could contribute to the markedly low leakage rate.

According to the available randomized data, the strength of MIE/RAMIE is the lower postoperative morbidity, especially a reduced rate of pulmonary complications. A recent propensity score-matched comparison and meta-analysis concluded that RAMIE has significantly lower rates of pneumonia or pulmonary complications than laparoscopic MIE and should potentially be considered the standard technique for esophagectomy [31, 37]. In the present trial, the rates of postoperative pneumonia and anastomotic leakage were 19.5% and 13.2%, respectively, compared with 23% and 20%, respectively, in the international registry (out of the 331 fully robotic Ivor Lewis cases) [14].

Interestingly, the present analysis showed that key characteristics and complications such as operative time, blood loss, the rate of pneumonia, and anastomotic leakage can be further improved after a learning experience of 22 cases, which was the initial CUSUM-based learning curve plateau for all five centers. The CUSUM analysis was designed for detecting minor changes in datasets to visualize trends describing the learning curve [8]. Interestingly, in another German single-center analysis, also a case load of 22 was necessary to overcome the learning curve for RAMIE procedure [1]. A comparable number of cases for completion of the learning curve (20–24 cases) have been reported by other centers, especially for experienced robotic-assisted surgeons [10, 15, 32]. In contrast, MIE was usually coupled with longer learning processes with flat learning curves; 54–119 cases were reported to be required to reach a stable plateau [3]. Robotic-assisted surgery instead displays distinct steeper learning curves, likely due to special da Vinci surgical system training programs and the existing competence of most participating surgeons in MIE surgery [27]. The present study further confirms that single experienced surgeons can reach the plateau for RAMIE within a proctored program even earlier.

Prior experience in robotic-assisted surgery seems to be of high importance. Increased overall and pulmonary complications and reoperation rates were observed after the TIME trial setting, with excellent short-term outcomes after MIE implementations in nationwide practice. The authors concluded that this may reflect the completion of the MIE procedure by nonexpert surgeons in a nonstandardized fashion outside of high-volume centers [20]. Therefore, recommendations toward RAMIE should be given after considering the center volume and experience of the leading surgeons.

The advantage of the study design is the multicenter setting with a uniform technique and the high quality of the data that was prospectively recorded and closely monitored. Alternatively, the data are limited by a heterogeneous set of lead surgeons and assistants in different centers, and minor modifications of the standard operative techniques were observed (e.g., insertion of jejunal feeding tubes or differences in the number of chest drains or the use of oral antibiotics on the day before the operation).

## Conclusions

In conclusion, the present high-quality multicenter registry data confirm that RAMIE is a safe procedure and can be reproduced with acceptable leak rates and promising short-term results in a multicenter setting. The learning curve is comparably low at approximately 22 cases for experienced surgeons and in a setting with interinstitutional proctoring.

## Supplementary Information

Below is the link to the electronic supplementary material.

Fig. S1. **Operative time for RAMIE including all centers.** (A) Abdominal part. (B) Thoracic part. hRAMIE procedures and operations with conversion were excluded. In A, the median operation time for the abdominal part in minutes is shown depending on chronological grouping of 15 cases (*p<0.001). In B, the median operation time for the thoracic part in minutes is shown depending on the chronological grouping of 15 cases (*p≤0.023). (PNG 6 kb)

High Resolution Image (TIFF 36 kb)

ESM 1 (PNG 6 kb)

High Resolution Image (TIFF 35 kb)

ESM 2 (DOCX 24 kb)
