# Supplementary figures and images for: Robotic-assisted minimally invasive Ivor Lewis esophagectomy within the prospective multicenter German da Vinci Xi registry trial
Source: Langenbecks Arch Surg. 2022 May 2;407(4):1–11. doi: 10.1007/s00423-022-02520-w (PMC9283356; doi:10.1007/s00423-022-02520-w)

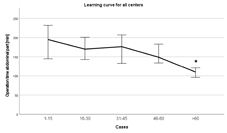

Supplement: Supplementary file 1 — Operative time for RAMIE including all centers. (A) Abdominal part. (B) Thoracic part. hRAMIE procedures and operations with conversion were excluded. In A, the median operation time for the abdominal part in minutes is shown depending on chronological grouping of 15 cases (*p<0.001). In B, the median operation time for the thoracic part in minutes is shown depending on the chronological grouping of 15 cases (*p≤0.023). (PNG 6 kb) [file 423_2022_2520_Fig4_ESM.png]

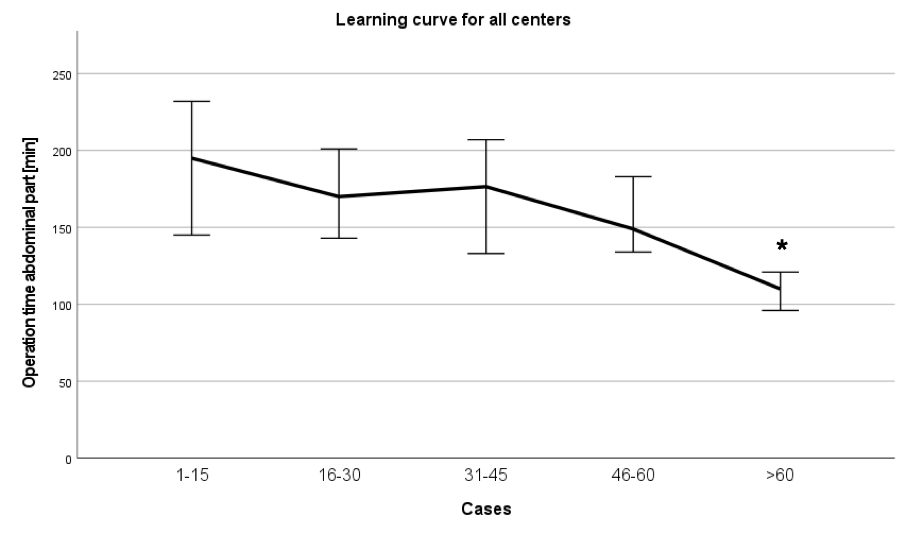

Supplement: Supplementary file 2 — High Resolution Image (TIFF 36 kb) [file 423_2022_2520_MOESM1_ESM.tiff]

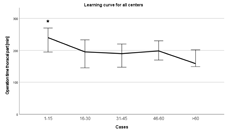

Supplement: Supplementary file 3 — (PNG 6 kb) [file 423_2022_2520_Fig5_ESM.png]

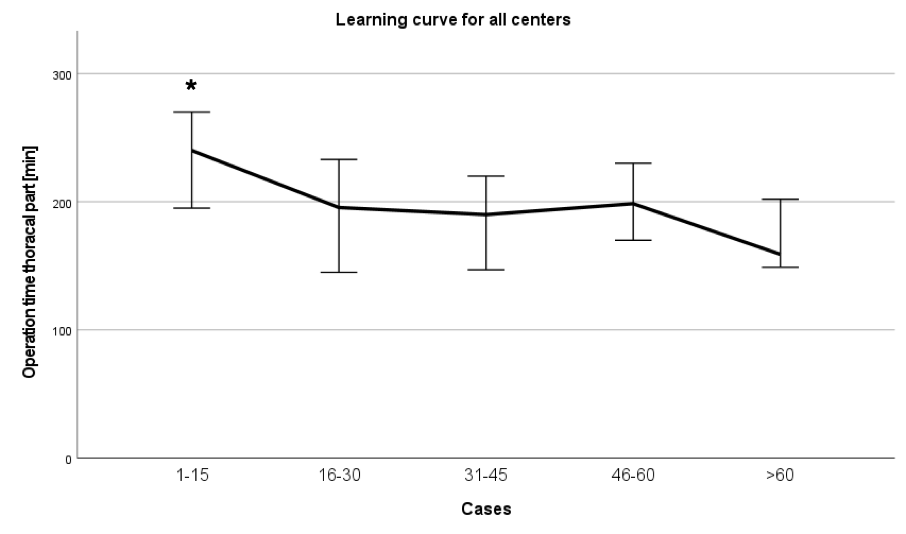

Supplement: Supplementary file 4 — High Resolution Image (TIFF 35 kb) [file 423_2022_2520_MOESM2_ESM.tiff]
