# Supplementary material for: Robotic-assisted minimally invasive Ivor Lewis esophagectomy within the prospective multicenter German da Vinci Xi registry trial
Source: Langenbecks Arch Surg. 2022 May 2;407(4):1–11. doi: 10.1007/s00423-022-02520-w (PMC9283356; doi:10.1007/s00423-022-02520-w)
Supplement: Supplementary file 5 — (DOCX 24 kb) [file 423_2022_2520_MOESM3_ESM.docx]

**Tab. S1.** Univariate analysis for anastomotic leak (n=220)

|  | **OR** | **95% CI** | **p-value** |
| --- | --- | --- | --- |
| **Age >65 vs. ≤65 years** | 0.83 | 0.374-1.824 | 0.394 |
| **Male vs. female** | 1.55 | 0.441-5.463 | 0.359 |
| **BMI >25 vs. ≤25 kg/m^2^** | 1.26 | 0.554-2.847 | 0.370 |
| **ASA ≥3 vs. ≤2** | 1.11 | 0.474-2.584 | 0.499 |
| **SCC vs. AC** | 1.17 | 0.411-3.326 | 0.474 |
| **Neoadjuvant treatment vs. none** | 0.92 | 0.350-2.423 | 0.518 |
| **RAMIE vs. hRAMIE** | 5.22 | 0.684-39.820 | 0.058 |
| **Conversion vs. no conversion** | 0.94 | 0.202-4.351 | 0.646 |
| **Extended lung resection vs. none** | 0.86 | 0.819-0.911 | 0.366 |
| **Extended lymphadenectomy vs. none** | 1.14 | 0.521-2.491 | 0.448 |
| **Circular stapler ≥28 vs. 25 mm** | 0.66 | 0.248-1.772 | 0.281 |
| **Operation time >425 vs. ≤425 min** | 1.29 | 0.592-2.843 | 0.326 |
| **Blood loss >200 vs. ≤200 ml** | 1.56 | 0.576-4.242 | 0.265 |
| **pT ≥3 vs. ≤2** | 1.13 | 0.511-2.517 | 0.456 |
| **pN ≥1 vs. 0** | 0.56 | 0.242-1.309 | 0.126 |
| **UICC ≥III vs. ≤II** | 0.51 | 0.218-1.197 | 0.086 |
| **Center cases >22 vs. cases 1-22** | 0.60 | 0.275-1.322 | 0.227 |
| **Surgeon cases >22 vs. cases 1-22** | 0.72 | 0.303-1.717 | 0.532 |
|  |  |  |  |

BMI: body mass index, ASA: American Society of Anesthesiologists, SCC: squamous cell cancer, AC: adenocarcinoma, OR: odds ratio, 95% CI: 95% confidence interval, Fisher’s exact test

**Tab. S2. Rates of key complications after minimally invasive esophagectomy in selected studies**

| Study | Operation^§^ | Number of patients (n) | Anastomotic leak n (per cent) | Pneumonia n (per cent) | Surgical site infection n (per cent) |
| --- | --- | --- | --- | --- | --- |
| Pointer 2020 | RAMIE | 350 | 55 (15.7) | 52 (14.9) | 19 (5.4) |
| Van der Sluis 2020 | RAMIE | 100 | 8 (8) | 12 (12) | 2 (2) |
| Mariette 2019 | Hybrid MIE | 102 | 11 (11) | 11 (11) |  |
| Van der Sluis 2019 | RAMIE | 54 | 13 (24) | 15 (28) | 2 (4) |
| Present study | RAMIE | 220 | 29 (13.2) | 43 (19.5) |  |

^§^Major type of operative technique
